# Fluorescence Characteristics of Coalbed Methane Produced Water and Its Influence on Freshwater Bacteria in the South Qinshui Basin, China

**DOI:** 10.3390/ijerph182412921

**Published:** 2021-12-08

**Authors:** Tao Jin, Qingjun Meng, Xiangdong Li, Lai Zhou

**Affiliations:** 1 School of Environmental Science and Spatial Informatics, China University of Mining and Technology, Xuzhou 221116, China; TS19160006A31@cumt.edu.cn (T.J.); xdli123@126.com (X.L.); zhoulai99@cumt.edu.cn (L.Z.); 2 Collaborative Innovation Center for Resource Utilization and Ecological Restoration of Old Industrial Base, Xuzhou 221116, China

**Keywords:** coalbed methane produced (CBM) water, fluorescence spectra, water environmental health, bacteria, fluorescent material

## Abstract

Production of coalbed methane (CBM) resources commonly requires using hydraulic fracturing and chemical production well additives. Concern exists for the existence of chemical compounds in CBM produced water, due to the risk of environmental receptor contamination. In this study, parallel factor method analysis (PARAFAC), fluorescence index, and the fluorescence area integral methods were used to analyse the properties of CBM produced water sampled from Shizhuang Block (one of the most active CBM-producing regions in the Qinshui Basin). A culture experiment was designed to determine the effect of discharged CBM produced water on microorganisms in freshwater. Water quality analysis shows the hydrochemistry of most water samples as Na-HCO_3_ type produced water of CBM appears as a generally weak alkaline (pH 8.69 ± 0.185) with high salinity, high alkalinity, and a high chemical oxygen demand (COD) value. Three individual components were identified by using parallel factor method analysis as humic-like components (C1), fulvic-like components (C2), and amino acid-like substances (C3). The fluorescence characteristic index comprehensively explains that the fluorescent substances in CBM produced water has the characteristics of a low degree of humification and a high recent self-generating source. The region integration results of characteristic peaks show that tyrosine-like and tryptophan-like materials account for more than 67% of fluorescent substances in CBM produced water. The addition of produced water from coalbed methane promotes the growth of freshwater bacteria, and this process is accompanied by the decrease of the proportion of fulvic acid, humic acid, and the increase of the proportion of soluble microbial metabolites. This paper proposes a convenient method for organic matter identification of CBM produced water and provides some theoretical support and reference for the improvement of CBM water treatment and utilization.

## 1. Introduction

To solve the problems of insufficient natural gas reserves, the greenhouse effect, and the safety of coal mines, coalbed methane (CBM) is considered an effective substitute [1]. Presently, the hydraulic fracturing technique is widely used in China’s coalbed methane mining [2]. The fracturing technique works on the principle of extracting CBM from coal seams by pumping water from wells located in the coalbed. The hydrostatic pressure is reduced, allowing methane gas to flow up to the wellbore surface, where extraction is less complicated [3]. This process generates a large amount of CBM produced water. The chemical composition of the produced water is variable [4]. It is determined by the chemical composition of the local formation water and by chemicals added from natural gas extraction. Organic substances in produced water can originate from the formation water, the solid phase associated with the formation water (coal or shale), oil present in the formation, and organic chemicals added during production. Chemicals added during production include those used for hydraulic fracturing, and other purposes [5,6] (e.g., thickeners, crosslinkers, pH adjusters, fungicides, clay stabilizers and demulsifiers).

Previous work on produced water from the typical large-scale coalbed methane producing area (America and Australia) indicated that extractable organic compounds consisted of many different substances, including polycyclic aromatic hydrocarbons (PAHs), heterocyclic compounds, other aromatics, phenols, and substituted phenols, long-chain fatty acids, and higher alkanes up to C25 [7,8,9]. No single producing well contained organic compounds in concentrations that would exceed Australian drinking water guidelines, or USEPA and WHO drinking water guidelines. However, care should be exercised in the disposal and release of produced waters containing these organic substances into the environment because of the potential toxicity of these substances.

Presently, there have been many studies on the impact of coalbed methane-produced water on environmental media and receptors after being discharged into the environment. Ganjegunte [10] evaluated the impact of land application of CBM produced waters on soil chemical properties northwestern of the Wyoming Powder River Basin. Soil chemical analyses indicated that electrical conductivity of soil saturated paste extracts (EC) and sodium adsorption ratio of soil saturated paste extracts (SAR) values for irrigated sites were significantly higher (*p* < 0.05) than control plots in the upper 30 cm soil depths. Vance [11] found that the species evenness of most non-CBM produced water irrigation areas is higher than that of CBM formed water irrigation areas; simultaneously, Shannon and Simpson diversity indexes of most non-CBM produced water irrigation areas are higher than those of irrigation areas. In additional research about the acute toxicity of CBM produced water to aquatic animals. Zhang measured the activities of antioxidant defense enzymes (SOD, CAT, and POD) in the liver and gill tissue of Carassius auratus and found that in comparison with the liver tissue, gill may be the major responsive organ of toxicity of CBM water in [12]. From a comprehensive perspective of environmental protection, the impact on the discharge environment of CBM produced water and its corresponding evaluation is a critical issue.

In countries such as China where CBM production developed relatively late, produced water disposal methods are immature: produced water is usually discharged into lined or unlined impoundments, into the field for agricultural use, into nearby water bodies [13]. CBM produced water has attracted increasing concerns about its potential ecotoxicological effects and ubiquitous occurrence in the aquatic environment [14]. However, knowledge about CBM produced waters’ influence on the bacteria community, which, as a consumer, is an essential component of freshwater, providing a valuable self-purification ecosystem service [15,16], remains largely unknown.

Compared with the analytical chemistry method with complex pretreatment, a convenient and rapid method is needed to identify the composition characteristics of organic matter in coalbed methane production water. Three-dimensional excitation-emission matrix fluorescence spectroscopy (henceforth referred to as 3DEEMs) is in common practice in pollutant identification, environmental monitoring, and pollution traceability due to its high sensitivity and lack of sample structure degradation [17,18,19]. Presently, the traditional peak method, fluorescence regional integration (FRI), fluorescence index (FI), etc. are the common uses of 3DEEM data application methods for judging water pollution sources, organic structure, and maturity indicators in water bodies [20,21,22]. In recent years, the apparent advantages of spectral characteristics have commonly been utilized in the identification of DOM in lakes and rivers. However, this method is seldomly reported in the study of DOM of CBM produced water.

The objectives of this study were to (1) identify organic pollutants in typical CBM production areas of three-dimensional fluorescence spectra, (2) evaluate the characteristics and sources of the organic matter in CBM, and (3) explore the effect of CBM drainage on freshwater bacteria.

## 2. Materials and Methods

### 2.1. Geological Setting of the Study Area

Qinshui Basin is one of the most active basins in southeast of Shanxi Province China for CBM exploration. The geographic coordinates are approximately 35° N, 112°00′ E, and 917 to 1322 m above sea level. The study area’ locations in the Shizhuang region of the southern Qinshui Basin, with primary coal-bearing strata include the Permian Shanxi and Carboniferous Taiyuan formations [23]. The hydraulic aquifers connected to the coal seam in the study area includes a sandstone aquifer in the Shanxi Formation and a limestone aquifer in the Taiyuan Formation, neither of which outcrops within the studied area [24].

### 2.2. Sample Collection and Water Quality Analysis Method

**Figure 1:**
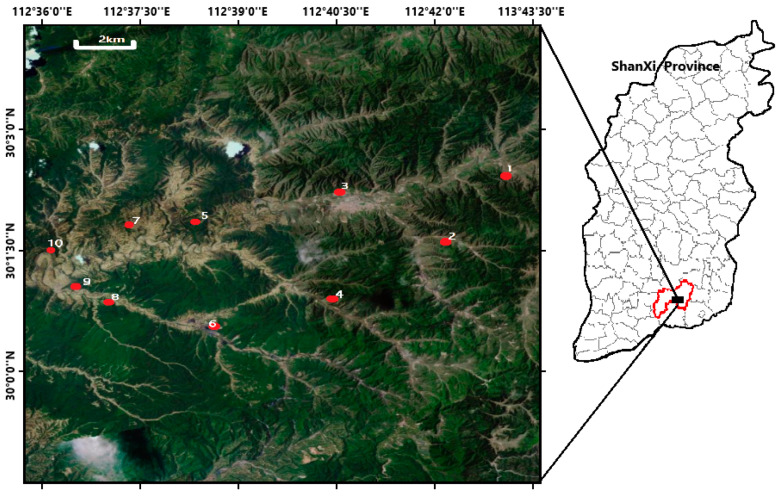
The 10 CBM wells sampled in this study locate in small folds developed in the southern slopes of Shizhuang (black block) in QinShui Basin (red circle).

Ten sampling points were arranged along with one of the small folds developed in the southern slopes of Shizhuang. They are all located in CBM wells with stable gas and water production, and their produced water belongs to the same aquifer (Figure 1). At each location, triplicate water samples were carefully collected in 5-L sterile polypropylene bottles. All water samples were transferred immediately to the lab and stored at 4 °C before determination within one week.

The pH and electrical conductivity (EC) values for CBM water and soil samples were determined using pH and EC electrodes (Shanghai INESA, FE30 andA30168). We filtered the water sample with a 0.45 μm glass fiber filter (Fisher, Waltham, MA, USA), the filter membrane and the filtrated water were dried at 105 °C to constant weight. The quality difference of the membrane and solid mass in filtrate were total dissolved solids (TDS) and suspended solids (SS), respectively [25]. Total alkalinity was measured by gran titration [26] using a semi-automatic titration system (AS-ALK2, Apollo SciTech, Newark, DE, USA). Total hardness was measured by ethylenediamine tetraacetic acid (EDTA) titrations [27]. Chemical oxygen demand (COD) was measured through potassium dichromate oxidation-spectrophotometric method, by using a multi-parameter water quality analyzer (Lianhua, 5B-3B (A)). Main ions including, Cl^−^, SO_4_^2−^, PO_4_^3−^ were determined by ion chromatograph (ECOIC Metrohm, equipped with an electrochemical detector and AS14 column); K^+^, Na^+^, Ca^2+^, Mg^2+^ were determined by inductively coupled plasma–optical emission spectroscopy (Perkin Elmer Optima 4300 ICP-OES); CO_3_^2−^, HCO^3−^ were determined by chemical titration.

Data statistics (mean, standard deviation, and ANOVA) were completed using SPSS25.0 software.

### 2.3. Fluorescence Features Test

**Measuring parameters**

The three-dimensional fluorescence spectrum analysis of water samples was carried out busing a synchronous absorption three-dimensional fluorescence scanning spectrometer (HORIB Aqualog-UV-NIR 800C). The main measurement parameters were as shown: excitation light source 150 xenon arc lamp; photomultiplier tube voltage 700 V; excitation wavelength (Ex) 231–450 nm; emission wavelength (Em) 248–827 nm; slit width 3 nm; scanning degree 2400 nm/min. Before being tested, all the water samples were filtered through a 0.45 μm glass fiber filter. The intensity of all excitation-emission spectra was normalized by dividing the integrated intensity area of the Raman water curve at 350 nm wavelength excitation from the 370−450 nm emission wavelength range. Data analysis included a correction due to water-scattering by subtracting the signal of a blank Milli-Q water sample analysed under the same conditions.

**Calculation method of excitation emission data**

Chen [28] first proposed fluorescence regional integration (FRI). It is based on the traditional peak method. According to the research purpose, the three-dimensional fluorescence spectrum is artificially divided into different regions, and the change of the fluorescent substance is quantitatively characterized by calculating the volume percentage of a given region. The specific normalized volume integration process is expressed as follows [29]:

(1) $$\varphi_{i,n}=MF_{i}\varphi_{i}=MF_{i}\iint_{exem}I(\lambda_{ex}\lambda_{em})d\lambda_{ex}d\lambda_{em}$$

(2) $$\varphi_{T,n}=\sum_{i=1}^{5}\varphi_{i,n}$$

(3) $$P_{i,n}=\varphi_{i,n}/\varphi_{T,n}\times 100\%$$

In the equation:

$\varphi_{i,n}$—Integral standard volume of the fluorescence region;
$FM_{i}$—Multiplication factor, which represents the reciprocal of the ratio of the area of a certain fluorescence integration area to the area of the total fluorescence integration area;
$\varphi_{i}$—Volume integration of the fluorescence, au·nm^2^;
$\lambda_{ex}$—Excitation wavelength, nm;
$\lambda_{em}$—Emission wavelength, nm;
$\varphi_{i}$—Volume integration of fluorescence, au·nm^2^;
$\varphi_{T,n}$—Total fluorescence area integration standard volume;
$P_{i,n}$—percentage of integral standard volum of the total fluorescence integration region;
$I\left(\lambda_{ex},\lambda_{em}\right)$—Fluorescence intensity corresponding to excitation wavelength or emission wavelength, au.

Chen et al. divided the entire spectral range into 5 regions, which are the tyrosine region, tryptophan region, fulvic acid region, soluble microorganism product (SMP) region, and humic acid region. Although researchers can perform different fine-grained divisions of the spectral region according to different research purposes, the basic regions are the same. This experiment uses Chen’s region division.

The quantified fluorescence intensity and content of different substances could be obtained by parallel factor analysis (PARAFAC) modeling, which is achieved by using the 3dEEM toolbox of MATLAB (R2018b). The equation is shown as follows [30]:

(4) $$\begin{array}{c} x_{ijk}=\overset{N}{\underset{n=1}{\sum }}a_{in}b_{jn}c_{kn}+e_{ijk}\\ i=1,2,\ldots ,I;j=1,2,\ldots ,J;k=1,2,\ldots ,K \end{array}$$

In this equation, *x_ijk_* is a ternary trilinear data matrix with a fraction or factor number n, that is, the constituent element of cubic matrix X (IxJxK); *a_in_*, *b_jn_*, and *c_kn_* represent the elements of component Matrices A, B, and C with clear physical meaning with sizes of *_ixn_*, *j_xn_* and *k_xn_* respectively, and *e_ijk_* is the component element of residual cubic Matrix e (ixjxk). *N* is the number of factors required to correctly fit the trilinear model, which is a fraction in the study of component decomposition. In this study, EEM results of 10 CMB samples were immediately put into this modeling. The reasonability of our PARAFAC modeling results was validated by the kernel consistency function analyses. Fluorescence indices (FI, BIX, and HIX) could be acquired based on the EEM-PARAFAC results. More details about these indexes can be found as followed.

The fluorescence index (*FI*_370_) is widely used to distinguish the main source of humic acid in water. It is defined as the ratio of the fluorescence intensity at the wavelength of 450 nm to the fluorescence intensity at the wavelength of 500 nm, when the excitation wavelength is of 370 nm. When *FI*_370_ ≥ 1.9 means endogenous, indicating that the main source of fulvic acid in water is a microbial source; and when the indicator *FI*_370_ ≤ 1.4, it is exogenous, indicating that the main source of fulvic acid in water is a terrestrial source [31,32,33].

(5) $$FI_{370}=\frac{FI_{450}}{FI_{500}},\;\lambda_{Ex}=370\;\mathrm{nm}$$

The biological index (*BIX*) has a direct relationship with the in-situ microbial activity intensity in water. It is defined as when the excitation wavelength was at 310 nm, the ratio of the fluorescence intensity at an emission wavelength of 380 nm to the maximum fluorescence intensity in the wavelength range of 420–435 nm. When the *BIX* value is greater than 1, it indicates that there is a strong microbial activity in the water body, and when the BIX value is lower (0.6~0.7), it indicates that the proportion of dissolved organic matters derived from microorganisms in the water body is low [32].

(6) $$BIX=\frac{FI_{380}}{FI_{Max420\sim 435}},\;\lambda_{Ex}=310\;\mathrm{nm}$$

In order to further reduce the filtration effect, humification index (HIX) was defined as the ratio of the fluorescence intensity at the excitation wavelength of 255 nm and the sum of the fluorescence intensity at the excitation wavelength of 435–480 nm to the sum of the fluorescence intensity at the excitation wavelength of 300–345 nm and the fluorescence intensity at the wavelength of 435–480 nm. In the range of 0–1, the *HIX* index increased with the increase of the DOM aromatization degree [34].

(7) $$HIX=\frac{\int \left(435,480\right)}{\int \left(300,345\right)+\int \left(435,480\right)}+\lambda_{Ex}=255\;\mathrm{nm}$$

### 2.4. Bacterial Growth Experiment and Drainage Simulation Experiment

#### 2.4.1. Freshwater Bacterial Growth Experiment

Luria–Bertani (LB) medium was prepared to provide essential nutrition for the proliferation of bacteria [35]: with 10 g of tryptone, 5 g of yeast extracts, and 5 g of sodium chloride per 1 L of distilled water. Groups under each experimental condition, LB broth was diluted into 10%, adjusted pH value between 7.4 and 7.6 by phosphate.

To reveal the impact of CBM produced water enrichment on the bacterial community in fresh water, CBM produced water in different proportions was spiked into 250 mL conical flasks containing liquid medium to reach a final concentration of 0% to 100%, with 10% intervals respectively. After sterilization, added 2 mL of Jinghu lake (a natural waterbody in Xuzhou city) water to the above series of culture solutions as a bacterial resource. Blank control is set up to correct the turbidity, colour, and sediment of the CBM produced water. The sample and the blank control are both made in 3 parallels. Placed these culture solutions after inoculation in a constant temperature incubator and cultured at 22 ± 2 °C, 24 h. UV-Vis Spectrophotometric method was used to measure the 24-h growth influence (reflected by absorbance OD600) [36] on bacteria content in natural water samples which were added CBM produced water at different concentrations.

#### 2.4.2. Drainage Simulation Experiment of CBM Produced Water

The Jinghu lake water was selected as the natural water. The CBM produced water from Shizhuang was seen as the raw water. The experimental group contained CBM produced water with percentages of 0%, 25%, 50%, and 100%, respectively, and were mark as W, X, Y, and Z. They were placed in a 2 L beaker respectively, in a room under normal light and temperature conditions, a 24-day drainage simulation experiment was performed. The CBM produced water did not need germicidal treatment. The bacteria in each water sample were measured every 3 days for a total of 24 days. The corresponding water samples after 24 days were labelled as W24, X24, Y24, and Z24. The plate count method was applied to determine the total number of bacteria in each water sample.

The total number of bacteria in the water samples was detected by the plate counting method. Experimental data were analyzed by SPSS 25.0 software.

## 3. Results and Discussion

### 3.1. Water Quality Analysis of CBM Produced Water

Water quality analysis of CBM produced water shows in Table 1. The produced water in the study area is generally weak alkaline, pH between 8.34 and 8.98. The high COD value, 2510 ± 170 mg/L, indicates that there is miscible oil related to shale gas or chemicals added in other production activities related to exploitation in the produced water. The TDS of the water sample is between 890–3380 mg/L, with an average of 2102 mg/L. The TDS values of specific sampling points differ greatly. This is due to the difference of sedimentary layer structure at each sampling point, or the fracturing work of a few wells that has destroyed the primary aquifer [37]. COD, TDS, and SS all exceed the emission standard for pollutants from the coal industry [38] to varying degrees.

**Figure 2:**
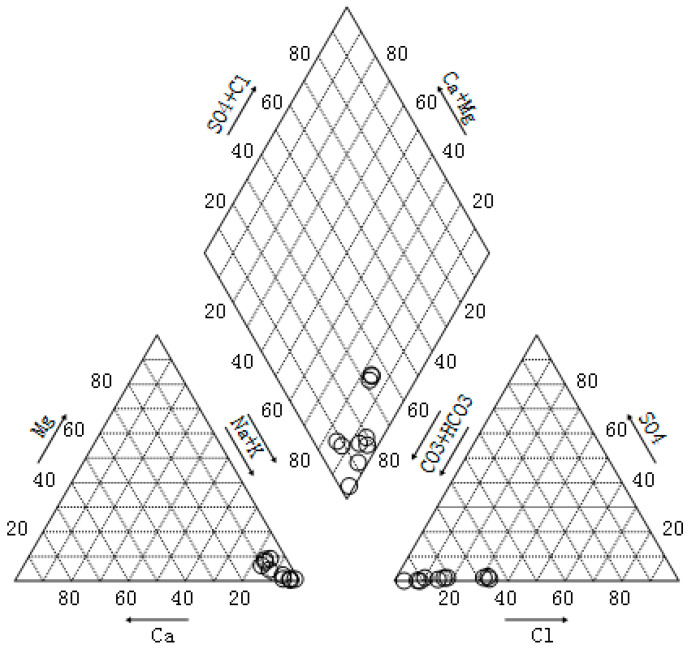
Hydrochemistry type Piper graph of CBM produced water (the eight ion types are Na^+^, K^+^, Ca^2+^, Mg^2+^, Cl^−^, SO_4_^2−^, HCO_3_^−^, CO_3_^2−^).

The main cations in the CBM produced water are Na^+^ and K^+^, their content accounts for 89.2% of the total cations. The main anions are CO_3_^2−^, HCO_3_^−^ and Cl^−^, which account for more than 97% of the total anions. The data confirms that Qinshui Basin belongs to continental coal deposits, are Na-HCO_3_ water types (Figure 2) similar to shallow groundwater formations of Na-HCO_3_ to Na-HCO_3_-SO_4_ type waters [39,40]. This aligns with the research conclusions made by Zhang [41] on the chemical characteristics, types, and genetic mechanisms of coalbed methane-produced water in the Qinshui Basin. The Na-HCO_3_ type water results from the long-term internal circulation of stratum water and the long-term rock-water interaction, which reflects that the CBM produced water in the study area originates from a semi-closed and open hydrogeological environment with free alternate water [42].

Beside these major anions and cations, the CBM produced water also contains small amounts of Ca^2+^, Mg^2+^, SO_4_^2−^, and PO_4_^3−^ ions. The sodium adsorption ratio (SAR) of produced water was calculated as follows [43]:

(8) $$SAR=\frac{Na^{+}}{\sqrt{\frac{Ca^{2+}+Mg^{2+}}{2}}}$$

where [ion] represent milliequivalent concentrations (meq/L) of the respective ions.

**Figure 3:**
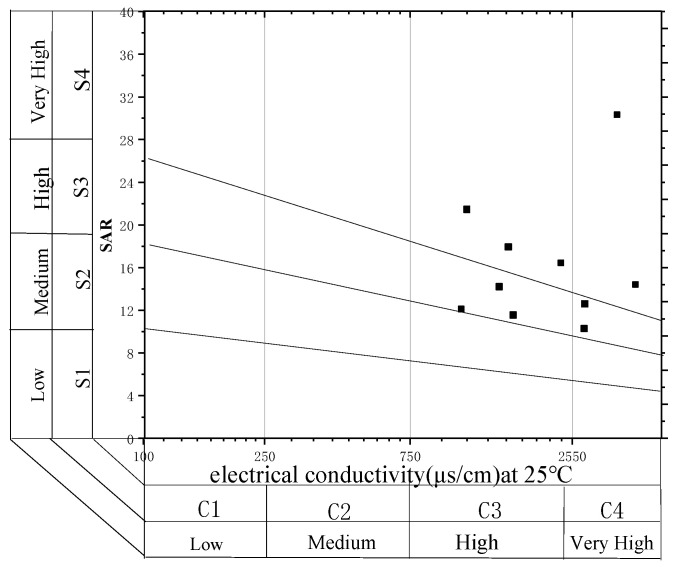
Salinity diagram of CBM produced water. According to the US Salinity Laboratory, salinity is expressed in terms of electrical conductivity and divided into four categories: low in salt (C1), salty (C2), highly saline (C3), and extremely saline (C4). The SAR value of the water is also divided into four categories: low (S1), moderate (S2), high (S3), and extremely high sodium content (S4).

The SAR of the produced water ranges from 18.29 meq/L up to 75.83 meq/L, with an average of 34.61 meq/L. The produced water in the study area is mostly with highly sa-line and high sodium levels, to highly saline with extreme sodium levels (Figure 3). The water cannot be used to irrigate the soil directly and must be treated; otherwise, it will cause significant damage [44].

### 3.2. Fluorescence Characteristics of CBM Produced Water

**Figure 4:**
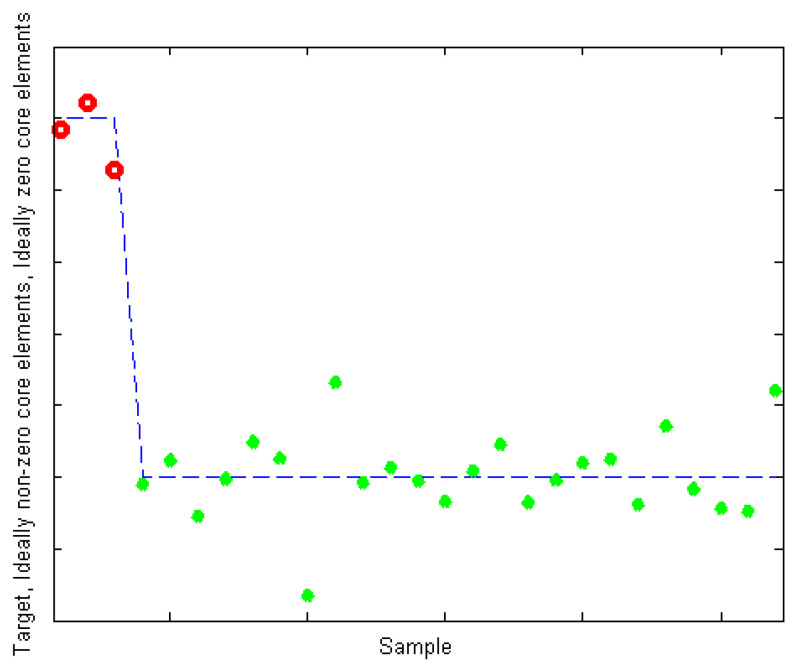
The red dot represent non-diagonal elements and the green dot represent diagonal elements, the degree of deviation of non-diagonal elements from 0 and the degree of deviation of diagonal elements from 1 are both smaller than those of other component number fitting cases. This result shows that the PARAFAC model with *N* = 3 is optimal.

After eliminating the effects of the scattering peaks, all samples formed a 10 × 125 × 75 fluorescence data array. A PARAFAC model of the data array was established to analyze the components of the fluorescent substances in the samples. Figure 4 shows the case of the core array elements when the number of components N = 3. When the DOM component number of coalbed methane is 3, its kernel consensus function is 88%, and 78.77% of the fluorescent substances in the water and can be interpreted. Therefore, the selection of component number N in this analysis was reasonable for the analytical result of the PARAFAC method.

**Figure 5:**
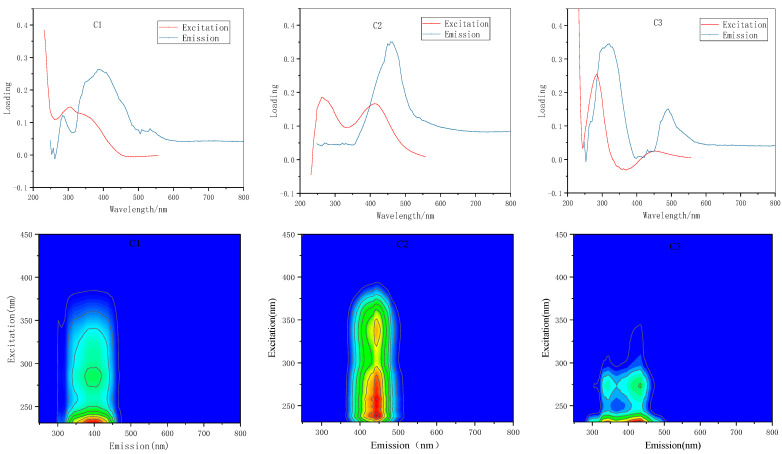
Three components identified by the PARAFAC model and Ex/Em loadings. (C1: humic-like components with carboxylic-like groups as primary compounds; C2: fulvic-like components with carboxylic-like and phenolic-like groups as primary compounds; C3: amino acids-like substances, easy to biodegradation and has strong correlation with human activities).

The three-dimensional fluorescence finger pattern and excitation emission spectrum of DOM in CBM produced water calculated by using PARAFAC method are shown in the Figure 5. C1 has a main excitation emission peak at Ex/Em = 231/384 nm, and a secondary peak at Ex/Em = 288/384 nm; C2 has excitation emission peaks at Ex/Em = 258,412/458 nm; C3 has an excitation emission peaks at Ex/Em = 219,279/329 nm. A peak of C1 is located in the range of long wave ultraviolet (UVA), belonging to UVA fulvic acid, and is close to the “M” peak [45], which is similar to the marine humus component [44]. C2 is a terrestrial humic acid component, and its peaks are close to “A” peak and “C” peak, respectively [46,47,48]. It is worth noting that in the three-dimensional fluorescence spectrum, the region where the peak is located may also have fluorescence peaks from artificial additives such as detergent and brightener [49,50], which should be distinguished from terrestrial humus. The indicative effect of these substances on additive pollution of coalbed methane wells still needs to be studied. C3 can be classified as a tryptophan-like component in proteins, similar to the “T” peak5 [51], which is mainly produced by biological activities of water body, with a high degree of freshness, which can well indicate the autogenetic process of water body [52,53]. The contents of C1, C2 and C3 respectively accounted for 42.03%, 33.04% and 24.93% of the total fluorescence components.

**Figure 6:**
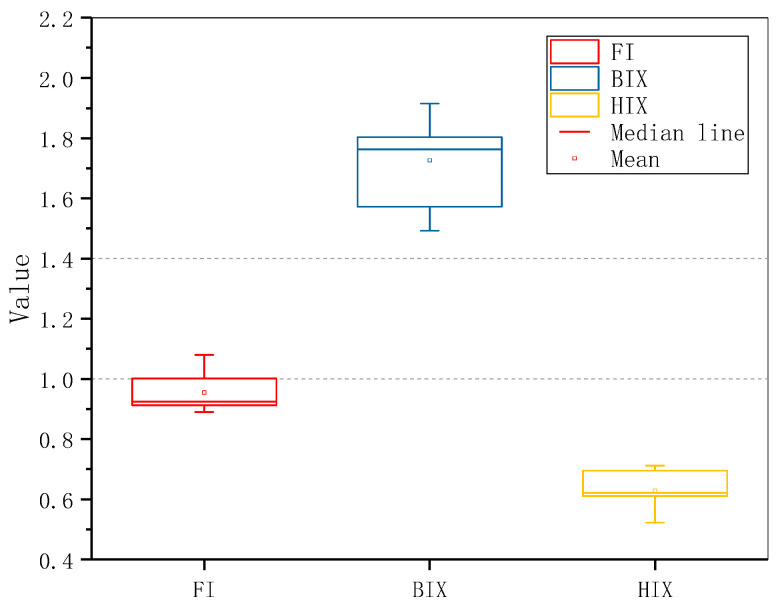
Box charts of FI, BIX and HIX of 10 CBM produced water samples. The whisker line represents 1.5 times the inter-quartile range, from which can judge that the dispersion of fluorescence index of each sample is low.

As shown in Figure 6, FI values of each point of CBM produced water ranged from 1.38 to 0.889, with an average value of 0.984. Thus, indicating that the primary source of humus in CBM produced water was a terrestrial source. BIX ranged from 1.49~1.91, average value 1.72, exhibiting that DOM in samples has a powerful source of microbial activity. This can be explained by the formation mechanism of coalbed methane: coal-bed methane generated by thermogenic or biogenic ways [54]. In the process of transformation, substances produced in the methanogenesis of microorganisms, such as oligomer monomer long-chain fatty acids, alkane, small molecules, aromatic hydrocarbons, organic acids or alcohols, are constantly produced, consumed and accumulated by microorganism in the occurrence environment. Forming the obvious characteristics of biological sources [55]. HIX values ranged from 0.522 to 0.712, with an average value of 0.628. The aromatization degree of CBM produced water is low [56]. Because as the maturity of CBM increases, the structure of coal changes from a heterocyclic macromolecule to a small molecule compound and finally to methane, so the aromatization degree of DOM in water produced with CBM is also low [57,58].

### 3.3. Bacterial Culture Experiment Results

**Figure 7:**
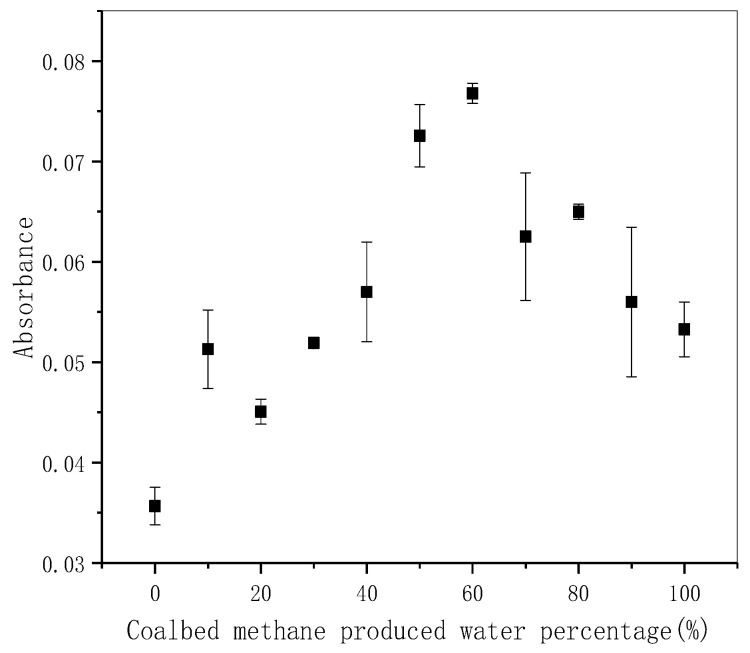
The 24 h-cultivation-effect of CBM produced water of different concentration on bacterial growth (the greater the absorbance value, the greater the density of bacteria in the culture solution, and the length of the error bar represents the uncertainty of the data).

In short time culturing (0–24 h), the different bacterial absorbance values of the samples with different concentrations of CBM produced water are shown in Figure 7. Under experimental conditions, the growth promotion effect reached the maximum at about 60% concentration, and the absorbance of the sample in this concentration increased by 1.14 times compared with the natural water. In the group where CBM produced water concentration is higher than 60%, the growth of bacteria is still under promoted, but the influence extent is relatively weakened. Because of the increase of the concentration of CBM produced, organic matter and other nutrients increases simultaneously, salinity and metal ion concentration also increase, which inhibits the proliferation of bacteria.

**Figure 8:**
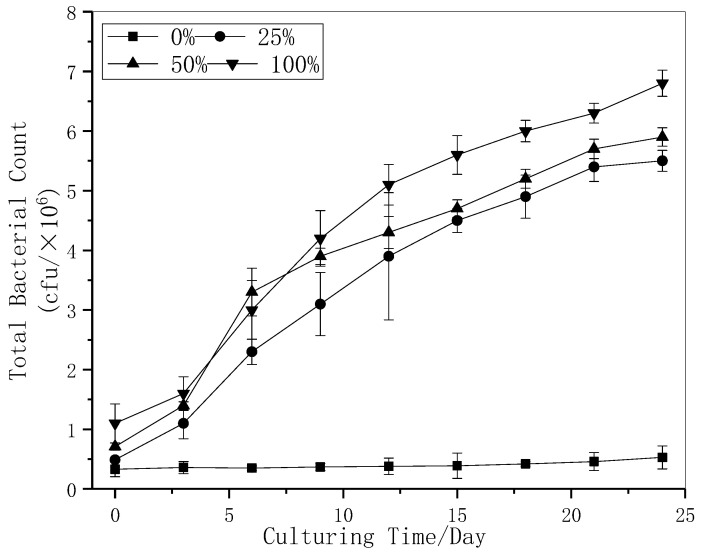
Variation curve of total bacteria in long-term culture experiments under different CBM produced water concentrations. Error bars indicate standard errors (n = 3).

Under the condition of adding different proportions (10 water samples mixed in equal proportion then diluted to 0% 25%, 50%, 100%) of CBM produced water into natural water, measuring the total amount of bacteria cultured in 24 days to study the effect of CBM produced water on total bacteria. The variation curve of total bacteria are shown in Figure 8.

The bacterial growth curves of the experimental group with the concentration of 25%, 50% and 100% were fit to the logistic growth model (Peter D.et al. 2017), and the fitting curves obtained (within 95% confidence interval) were as follows:

$y_{25\%}=5.533/(1+7.142\times e^{-0.24t}$), R^2^ = 0.993; $y_{50\%}=5.668/(1+4.839\times e^{-0.249t}$), R^2^ = 0.966; $y_{100\%}=6.641/(1+5.315\times e^{-0.236t}$), R^2^ = 0.993, respectively. This indicates that in the limited environment where CBM produced water is added to—the bacterial community—it can also proliferate normally. On the 24th day, the number of bacteria in the water with different proportions of CBM was 5.5 × 10^6^, 5.9 × 10^6^ and 6.8 × 10^6^ CFU, respectively, which was 12.8~15.8 times of that in the control group. One-way ANOVA was used to test whether the added content of CBM produced water has a significant effect on the growth of bacteria in the culture medium. The analysis result was F = 222.973, *p* < 0.01, and is considered that the percentage of CBM content has a significant effect on the promotion of bacterial growth.

**Figure 9:**
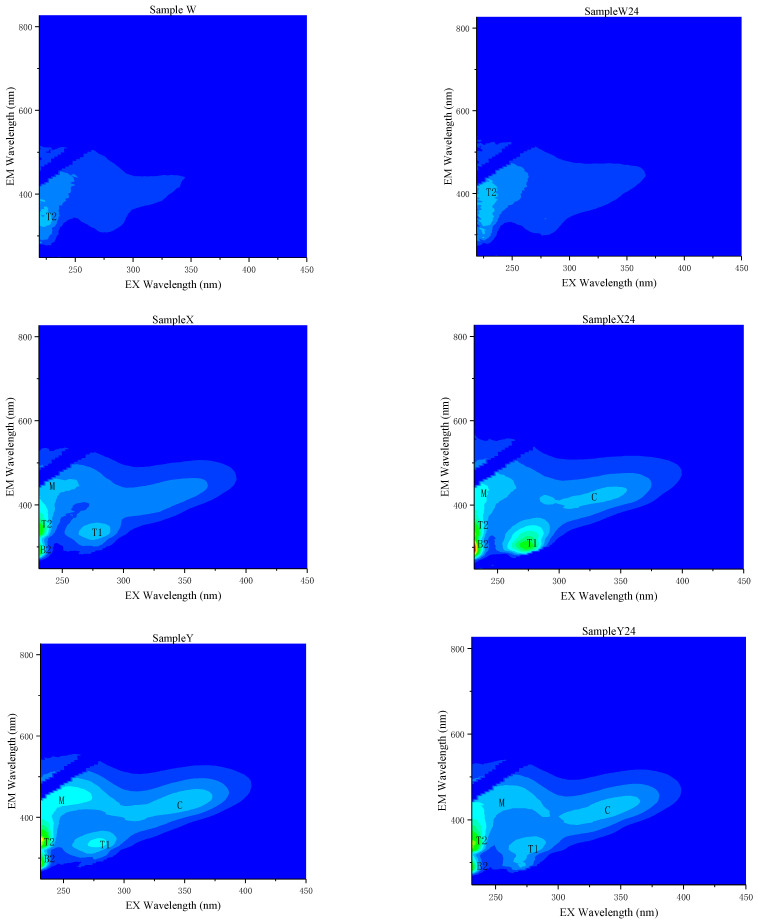

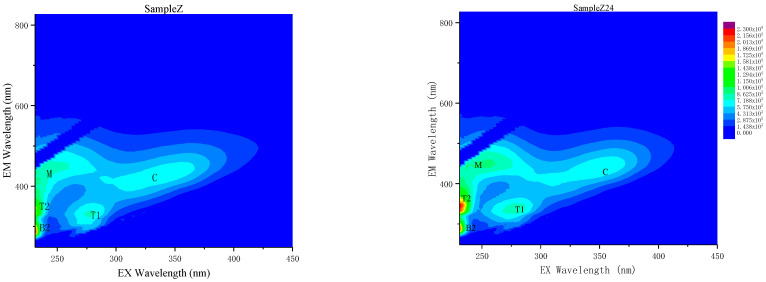
3DEEM contour of a natural water Sample W, W 24 and samples added with different proportions of CBM produced water (the color bar represents the change of spectral intensity, where all eight figures are under the same color bar).

Meanwhile, analyzing the 3D fluorescence spectrum of the initial water sample (Day 0) which was added with different proportions of CBM produced water. As well as and of the corresponding samples after the 24-day lasting simulation experiment. Their respective fluorescence characteristics, the fluorescence peak, the fluorescence intensity, and the changes are shown in Figure 9.

The natural water Sample W has two weakened contours fluorescence peaks, peakT2 (low excitation light tryptophan, Ex/Em 225–250/339–350, max fluorescence intensity 4488) and PeakT1 (high excitation light tryptophan, Ex/Em 270–290/315–365, max fluorescence intensity 2072) [22,59]. After 24 days, the water sample W-24 showed an obsolete fluorescence intensity change. We can preliminarily understand that the dissolved organic matter in the natural water body selected by the experiment is mainly a protein-like fluorescent substance from a biological source.

Combined with the analysis conclusion in Section 3.2, from the 3D fluorescence spectra result of water Sample Z, we learned that the raw CBM produced water has five fluorescence characteristic peaks, which are T2 peak (low excitation light tryptophan, max fluorescence intensity 18,940) and T1 peak (high excitation light tryptophan, max fluorescence intensity 7049). According to the previous studies, T1 and T2 represent free amino acids or bound amino acids in proteins. Their fluorescence characteristics are as free tryptophan. They are widely present in marine and terrestrial waters, which are derived from the life metabolism of in situ microorganisms, and can represent undegraded protein or relatively low degradation peptides in water. B2 peak (Ex/Em 220–230/290–310, max fluorescence intensity 16,587. The fluorescence characteristics of this peak are comparable to free tyrosine. Its source is also related to the life activity of the in situ organism. Indicating higher a degradation degree than amino acid [60]. C peak (Ex/Em300–370/400–500, max fluorescence intensity 6202) and M peak (Ex/Em230–280/400–500, max fluorescence intensity 7746) are both typical humus-like components [61,62].

After 24 days of experiments, the fluorescence intensity of amino acid substances in the water sample Z24 showed an increase: B2 peak max fluorescence intensity to 21,621, T2 peak max fluorescence intensity to 22,958, T1 peak fluorescence intensity to 8208. Red shifts were observed for Em of Peaks C due to the potential structural and conformational changes undergone by DOM molecules after biological and photo degradation. The types of fluorescence peaks in the experimental group that added 25% CBM produced water to natural water (X and X-24) and the 50% CBM produced experimental group(Y and Y24) shows the same variation trend, the only difference is the intensity of the fluorescence peaks.

**Figure 10:**
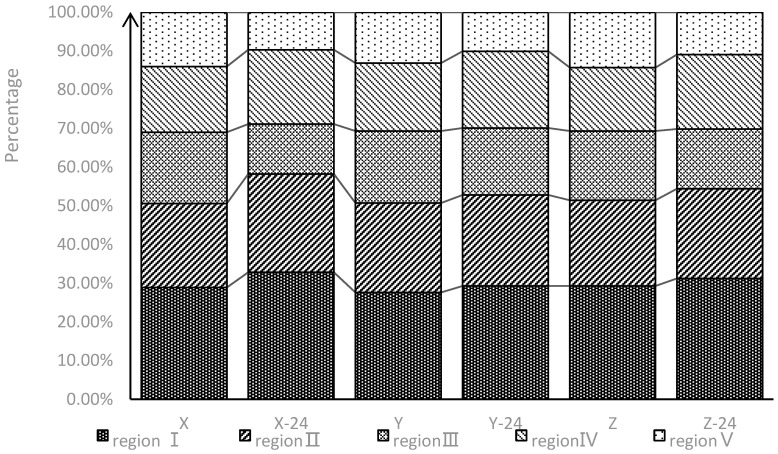
Percentage distribution of fluorescence regional integration results for Sample X, X-24, Y, Y-24, Z and Sample Z-24 (Regions I and II represent protein substances; Region III represents fulvic acid substances, Region IV represents soluble microbial products, and Region V represents humic acid substances).

Dividing the fluorescence spectrum into five categories and using the fluorescence regional integration (FRI) method to compare the component of fluorescent substances before and after 24 days. The result calculated by FRI is shown in Figure 10.

FRI distributions over five typical regions are shown before and after 24 days for different concentrations of CBM waste water. During 24 days of a drainage simulation experiment, the values of P_I,n_, P_II,n_, and P_Ⅳ,n_ increased from 28.6% to 31.3%, from 22.3% to 24.0%, and from 17.0% to 19.4%, respectively; the values of P_Ⅲ,n_, and P_V,n_ decreased from 18.3% to 15.2% and from 13.8% to 10.2%, respectively. The sum of P_I,n_, P_II,n_, and P_IV,n_ was from 67% to74%, and this result indicated that tyrosine-like and tryptophan-like materials were the main constituents for both before and after 24 days. The contents of fluorescent materials varied in the order tyrosine-like > fulvic-like > tryptophan-like > humic-like materials, according to their mean P_I–IV,n_ values. We can speculate that increases in amino acids was due to life activities, while humic substances decreased due to photodegradation and biological metabolism. The possible action process and mechanism of specific microorganisms should be the subject of further studies in the future.

## 4. Conclusions

The produced water of coalbed methane in Shizhuang District of the Qinshui Basin is mainly Na-HCO_3_ water type, which comes from the terrigenous sedimentary layer, and reflects that the CBM produced water in the study area originates from the semi-closed and open hydrogeological environment with free alternate water. The produced water of the coalbed methane appears to be a generally weak alkaline, with a pH value between 8.34 and 8.98, with high salinity, high SAR, and high COD value. Many organic and inorganic substances in the produced water may come from the gas coalbed depositional environment, aquifer interactions process, and anthropogenic activities such as fracturing production. This kind of CBM produced water is not suitable for irrigation or direct discharge, which possesses certain environmental risks.

Five main peaks, Peak T1 (Ex/Em 270–290/315–365 nm), Peak T2 (Ex/Em 225–250/339–350 nm), Peak B2 (Ex/Em 220–230/290–310 nm), Peak C (Ex/Em 300–370/400–500 nm) and peak M (Ex/Em 230–280/400–500 nm) were observed in 3DEEM contour. The average values of FI, HIX, and BIX were 0.984, 0.628, and 1.72, respectively. The fluorescent substance in the CBM produced water from this area has the characteristics of a low degree of humification and a high recent self-generating source. According to FRI analysis, the sum of P_I,n_, P_II,n_, and P_IV,n_ was more than 67%, indicating that tyrosine-like and tryptophan-like materials were the main constituents. Three PARAFAC components were identified by residual analysis (R > 0.9999). The C1 (Ex/Em 288/284 nm, 2288/384 nm), the C2 (Ex/Em 258/485 nm, 412/458 nm), and the C3 (Ex/Em 219/329 nm, 273/329 nm) were categorized as humic-like components with carboxylic-like groups as primary compounds, fulvic-like components with carboxylic-like and phenolic-like groups as primary compounds, and amino acids-like substances, easy biodegradation and has a strong correlation with human activities, respectively.

The experiment of bacterial growth influence for 24 h was carried out by adding different concentrations of CBM produced water. We found that the CBM produced water generally promoted the growth of bacteria, but there was no linear relationship between the promotion effect and the produced water concentration. Its promotion effect reached the maximum at about 60% concentration. In the drainage simulation experiment, we found that in the limited environment where CBM produced water is added to the bacterial community can also proliferate normally, bacteria in the water with different proportions of CBM reached 5.5 × 10^6^ CFU, 5.9 × 10^6^ CFU, and 6.8 × 10^6^ CFU correspondingly, which was 12.8~15.8 times of that of the control group. One method ANOVA was used to claim the added content of CBM produced water has a significant effect on the growth of bacteria in the culture medium. The fluorescence intensities increased from 7049, 18,940, 16,587 to 8208, 22,958, 21,621 during the drainage simulation experiment for Peaks T1, T2, and B2, respectively. Redshifts were observed for Em of Peaks C and due to the potential structural and conformational changes undergone by DOM molecules after photo-degradation and micro-degradation. Concisely, after the produced water of coalbed methane is discharged into the water body containing natural freshwater microorganisms. The biogenic fluorescent material peak is more obvious than the properties of macromolecular humus. It is easy to have degraded changes, and the relative content decreases.

## Figures and Tables

**Table 1:** Basic physical and chemical properties of water samples.

Sample	Total Hardness (mg/L)	Total Alkalinity (mg/L)	Electrical Conductivity (μs/cm)	pH	TDS (mg/L)	SS (mg/L)	COD (mg/L)
1	23.45 ± 0.27	997.43 ± 4.00	4107 ± 6	8.34 ± 0.01	3880 ± 17	246.3 ± 4.8	2680 ± 4
2	9.57 ± 0.43	735.23 ± 7.05	2340 ± 3	8.98 ± 0.02	2690 ± 10	326.8 ± 3.0	2458 ± 10
3	65.32 ± 0.92	543.23 ± 3.41	1573 ± 6	8.63 ± 0.01	1200 ± 10	349.4 ± 1.2	2430 ± 6
4	32.45 ± 1.44	456.77 ± 4.67	1150 ± 10	8.84 ± 0.02	1400 ± 12	308.6 ± 3.5	2558 ± 3
5	46.34 ± 1.10	677.96 ± 7.00	3570 ± 6	8.70 ± 0.02	2700 ± 44	338.4 ± 0.4	2460 ± 3
6	37.98 ± 1.66	934.22 ± 5.27	2789 ± 5	8.55 ± 0.03	2560 ± 17	230.3 ± 1.4	2498 ± 5
7	25.32 ± 2.36	718.97 ± 3.91	1630 ± 7	8.73 ± 0.03	1898 ± 17	328.5 ± 7.3	2520 ± 4
8	97.33 ± 2.28	567.89 ± 6.16	1103 ± 8.6	8.65 ± 0.01	890 ± 26	320.4 ± 4.4	2570 ± 4.7
9	46.34 ± 1.23	489.34 ± 1.91	1468 ± 6.2	8.83 ± 0.02	1180 ± 26	306.4 ± 2.7	2376 ± 2.0
10	48.22 ± 1.30	712.65 ± 4.31	2800 ± 8.5	8.96 ± 0.01	2350 ± 44	349.8 ± 3.4	2550 ± 4.2

## Data Availability

The data presented in this study are available on request from the corresponding or first author.
